# PCM and TAT co-modified liposome with improved myocardium delivery: in vitro and in vivo evaluations

**DOI:** 10.1080/10717544.2016.1253121

**Published:** 2017-02-06

**Authors:** Xin Wang, Hua Huang, Liangke Zhang, Yan Bai, Huali Chen

**Affiliations:** 1 Department of pharmaceutics, School of Pharmacy, Chongqing Medical University, Chongqing, P. R. China and; 2 Chongqing Research Center for Pharmaceutical Engineering, Chongqing, P. R. China

**Keywords:** PCM, TAT, co-modified, coumarin-6, myocardium delivery

## Abstract

In this study, PCM and TAT co-modified liposome was developed as a novel drug carrier for myocardium delivery with evaluation of its *in vitro* and *in vivo* properties. Liposomes containing fluorescent probe coumarin-6 were prepared by thin-film hydration. The PCM ligands specifically bind to the PCM receptors in the extracellular connective tissue of primary myocardium cells (MCs), while the TAT ligands functioned as a classical cell penetrating peptide to make liposomes internalized by MCs. The unmodified liposome (L), PCM-modified liposome (PL), TAT-modified liposome (TL) and PCM and TAT co-modified liposome (PTL) were prepared and characterized. The cellular uptake and intracellular distribution of various liposomes by MCs demonstrated that PTL had the best delivery capability. Peptide inhibition assay indicated that the uptake of PL could be inhibited by PCM. However, TAT could almost not suppress the uptake of TL. In addition, the CCK-8 experiments showed that liposomes had low cytotoxicity. *In vivo* fluorescent images of frozen sections and HPLC-fluorescence analysis further demonstrated that PTL had highest myocardium distribution. The results of this study demonstrated that PCM and TAT co-modifying could improve the myocardial targeting ability of liposome.

## Introduction

Myocardial ischemia (MI) is one of the most common causes of death in the world. It is a disease caused by coronary artery stenosis and by the lack of oxygen within cardiac muscles due to an imbalance between oxygen supply and demand. The conventional medical therapy is focused on the coronary-artery bypass graft surgery, drug eluting stents and anti-thrombosis (Won et al., 2014). Despite advances in clinical management, ventricular dysfunction and heart failure remain the major causes of morbidity and mortality following MI (Sutton & Sharpe, 2000). Compared to surgical treatment, drug therapy is more secure, convenient and with lower cost, but its curative effect is not good enough as it fails to accumulate in the lesion site effectively. Liposomal drug nanocarriers as attractive pharmaceutical delivery system, has expanded considerably over the past few decades. With active ligand modification, liposomes can enrich in the lesion to achieve the best effect of targeted therapy (Noble et al., 2014; Li et al., 2016). Ligand-targeted liposome as a novel drug-delivery system has been applied to the pharmaceutical research of the treatment of MI in recent years.

Anti-myosin 2G4 antibody (Liang et al., 2004), anti-cardiac troponin I antibody (Liu et al., 2014), anti-P-selectin (Scott et al., 2009) and 7-mer peptides (Dasa et al., 2015) were used to combine with liposomes to achieve myocardial targeting, and these studies have achieved fairly good results. PCM (WLSEAGPVVTVRALRGTGSW) is a much more specific ligand which is a 20-mer peptide obtained by phage display biopanning. PCM could selectively target primary cardiomyocytes, isolated PCM phage binds primary cardiomyocytes 180 times more avidly than control phages and phage displaying this peptide preferentially bind to cardiomyocytes when compared with a panel of other cell types (Barry et al., 1996; McGuire et al., 2004; Won et al., 2014). PCM has been used to modify bioreducible polymer carrier containing Fas siRNA for the potential treatment of cardiovascular disease (Nam et al., 2010), the study found that PCM-modified polymers can specifically bind cardiomyocytes and improving transfection efficiency. Therefore, we choose PCM as a specific ligand to modify liposome with a view to achieve better targeting properties.

The component of the cardiomyocytes that interacts with PCM is an extracellular matrix protein called tenascin-X. While PCM was able to deliver a therapeutic moiety to cardiomyocytes, its tenascin-X receptor might cause a down-regulation and/or saturation of expression, which might make it inefficient to construct an effective targeted delivery system of high cellular uptake rate (Warren et al., 1997; Sawant & Torchilin, 2012). To avoid the occurrence of such a situation, the transactivating transcriptional activator peptide TAT (YGRKKRRQRRR) was connected to the surface of the liposome as a co-modified ligand. TAT is a protein expressed by the LTR trans activating gene of HIV-1, the former study showed TAT could efficiently introduce various small molecules as well as large macromolecular therapeutics (Schwartz & Zhang, 2000). TAT ligand could promote the intracellular penetration through a nonspecific endocytosis and concentration-dependent way (Torchilin, 2008; Yuan et al., 2016). Moreover, it was reported that TAT and another specific ligand anti-myosin 2G4 antibody was used to connect with liposome as a dual-ligand delivery system to increase liposome accumulation in the ischemic myocardium (Ko et al., 2009).

Therefore, a kind of PCM and TAT co-modified liposome was designed to target primary myocardium cells (MCs) in the present study. Lipid materials were made up of SPC, CHO, DSPE-mPEG2000 and DSPE-PEG2000-MAL. PCM and TAT were conjugated covalently to the distal end of DSPE-PEG2000-MAL. Coumarin-6 was encapsulated as a ﬂuorescent probe for *in vitro* and *in vivo* study to confirm the targeting efficacy to myocardium. *In vitro*, the primary MCs uptake, intracellular distribution, peptides inhibition effect experiments and cytotoxicity assay were carried on to examine whether this drug-delivery system had advantages over simple liposome (L) and single ligand-modified liposome (TL and PL). *In vivo*, imaging of frozen sections of the mouse heart and high-performance liquid chromatography assay were operated to achieve the same purpose.

## Methods

### Materials

Soybean phospholipids (SPC), Cholesterol(CHO), DSPE-mPEG2000 and DSPE-PEG2000-Mal were all purchased from Shanghai Advanced Vehicle Technology L. T. D. Co. (Shanghai, China). PCM and TAT peptide with terminal cysteine (WLSEAGPVVTVRALRGTGSW-Cys and AYGRKKRRQRRR-Cys) were synthesized by GL Biochem Ltd. (Shanghai, China). Coumarin-6 was purchased from J&K Scientific LTD. (Beijing, China). Sephadex G-50 was purchased from Pharmacia Biotech (Uppsala, Sweden). 4′, 6-Diamidino-2-phenylindole (DAPI) was purchased from Beyotime Biotechnology Company, Limited. (Nantong, China). Cell Counting Kit-8 (CCK-8) was purchased from Dojindo (Xiongben, Japan).

Cell culture flasks, plates and centrifuge tubes were purchased from Corning Incorporation (Corning, New York, NY). Dulbecco’s-modified Eagle’s medium (DMEM, high glucose), fetal bovine serum (FBS), trypsin-EDTA (0.25%) and penicillin–streptomycin were purchased from Gibco (MA, USA). All the other reagents and chemicals of analytical grade were purchased from Chongqing Zhuonuo Biotechnology Ltd. (Chongqing, China).

Kunming mice (15 ± 2 g) and two-day-old Sprague-Dawley rats were purchased from Experiment Animal Center of Chongqing Medical University. All animal procedures and use of laboratory animals for this study were approved by the Experiment Animal Administrative Committee of Chongqing Medical University.

### Synthesis of DSPE-PEG2000-PCM and DSPE-PEG2000-TAT

DSPE-PEG2000-PCM and DSPE-PEG2000-TAT were synthesized in the aqueous solution. Briefly, DSPE-PEG2000-Mal was dissolved in chloroform and dried in a RE-52AA rotary evaporator (Yarong Biochemistry Instrument Company, Shanghai, China) in a round-bottom flask at 40 °C, the obtained thin film was kept in vacuum for over 2 h to remove the residual solvent. Then, the thin film was hydrated by Ultrapure water in a bath-type sonicator, then PCM peptide and TAT peptide in Ultrapure water were, respectively, added to the micelle solution (the molar ratio of peptide and DSPE-PEG2000-Mal was 1.2:1). The mixture was allowed to react at room temperature overnight, thus the DSPE-PEG2000-PCM and DSPE-PEG2000-TAT were obtained.

### Preparation of coumarin-6 loaded L, TL, PL and PTL

The liposomes containing coumarin-6 were prepared by the thin-film hydration method. Briefly, the different amounts of SPC, CHO, DSPE-PEG2000 (60:30:10, mol/mol) and coumarin-6 were dissolved in the mixed solvent of chloroform and methanol (V:V = 3:1), and the organic solvent was removed by rotary evaporation at 40 °C. Then, the obtained thin film was kept in vacuum for over 2 h. The thin film was hydrated in 2 ml pH 7.4 PBS by oscillation for 1 h at 37 °C, followed by a bath-type sonication until the lipid film was completely dissolved. The mixture was further sonicated by a probe sonicator in ice-bath (running 5 s, pause 5 s, 15 times). At this point, the unmodified ordinary liposome was prepared. For the preparation of TL, PL and PTL, simply add DSPE-PEG2000-PCM (3%) or DSPE-PEG2000-TAT (1%) or both to the obtained ordinary liposome after probe ultrasound, and then oscillating for 4 h.

### Characterization of various coumarin-6 loaded liposomes

The particle size and Zeta-potential of the L, TL, PL and PTL were determined by a Malvern Zetasizer Nano ZS90 (Malvern Instruments Ltd., UK). The morphology of TPL were measured by an H-600IV Transmission Electron Microscope (Hitachi, Japan). For the encapsulation efficiency (EE) of coumarin-6 loaded L, TL, PL and PTL, a 1 ml liposome suspension was passed through a Sephadex G-50 column in PBS (pH 7.4) solution, and the concentration of coumarin-6 were detected by an RF-5301 pc fluorospectrophotometry (*E*x = 465 nm, *E*m = 502 nm, Shimadzu Corp., Japan). The encapsulation efficiency was calculated based on the ratio of mean value after gel-filtration and mean value before gel-filtration.

### *In vitro* stability of PTL

*In vitro* stability experiment of PTL using PBS (pH 7.4) was performed by studying variations of particle size, zeta-potential, polydispersity index (PDI) and encapsulation efficiency during 30 d period. PTLs were placed at 4 °C in PBS (pH 7.4), particle size, zeta-potential, polydispersity index (PDI) and encapsulation efficiency were detected at day 0 and day 30, respectively. Besides, the *in vitro* stability of PTL in PBS, pH 7.4 containing 10% FBS and 50% FBS was performed by measuring turbidity variations. Briefly, PTLs were mixed with equal volume of 10% FBS or 50% FBS under 37 °C with gentle shaking at 50 rpm. Then, 100 μl of the sample was pipetted out onto a 96-well plate to measure the absorbance at 750 nm by a microplate reader (Thermo Scientific Varioskan Flash, Waltham, MA) at predetermined time points (1 , 2 , 4 , 8  and 24 h).

### Cytotoxicity of L, TL, PL and PTL

*In vitro* cytotoxicity of different liposomes were detected by Cell Counting Kit-8 (CCK-8). Briefly, MCs were seeded in 96-well plates at a density of 5 × 10^3^ cells/well and cultured at 37 °C for 24 h. 24 h later, cells were washed once with PBS and exposed to five serial concentrations of L, TL, PL and PTL without coumarin-6. After 24 h, the culture media was removed and 100 μl CCK-8 diluted in PBS (v/v = 10:90) was directly added to each well. Then, the cells were cultured continuously for 2 h at 37 °C. The absorbance was then read on a microplate reader (Thermo Scientific Varioskan Flash, Waltham, MA) at a wavelength of 450 nm. The experiment was carried out in triplicate and cell viabilities of the samples were expressed as the percentage of the control absorbance values without any treatments.

## *In vitro* uptake of coumarin-6 labeled L, TL, PL and PTL by MCs

### Primary culture of rat MCs

Two-day-old Sprague-Dawley rats were soaked into 75% ethanol solution until the skin became ruddy. Then, they were sacrificed immediately to obtain the hearts and were cleaned and snipped into small pieces of 1 mm^3^. Then, the small pieces were transferred into a 50 mL centrifuge tube and digested by 3–4 ml of trypsin–collagenaseIIsolution (w/w = 1:1) for 3–5 min in water bath at 37 °C. Then, the digested solution was transferred to DMEM medium supplemented with 10% fetal bovine serum. The digestion procedures of the abovementioned tissues were repeated until the tissues were disappeared. The cell suspension was centrifuged at 1400 rpm for 5 min and suspended twice in DMEM to clean the cells. Then, the cells were resuspended in culture medium and seeded in cell culture flask. After incubating for 45 min, most of the obtained fibroblasts were adherent, however, MCs were still suspended. MCs suspension was then transferred and seeded into 6-well plates. On the second day, MCs in 6-well plates were treated with 5-bromo-2-deoxy uridine (Brdu) to inhibit the growth of fibroblasts. In the next day, MCs were refreshed with new culture media and incubated for uptake experiments.

### Flow cytometry

MCs were seeded at a density of 30 × 10^4^ cells/well in 6-well plates and cultured at 37 °C for 24 h to allow cell attachment. After 24 h, the cells were washed with PBS and incubated with L, TL, PL and PTL diluted with FBS-free DMEM medium for 1 h at 37 °C, with final coumarin-6 concentration of 200 ng/ml. Then, the medium was removed and cells were washed three times with PBS. The cells were then harvested by trypsinization, centrifuged at 1400 rpm for 5 min and washed three times with cold PBS. Then, the cell samples were examined by flow cytometry using a FACScan (Becton Dickinson, San Jose, CA).

### Confocal laser microscopy

MCs were seeded in 24-well plates with the cover slips at the bottom at a density of 4 × 10^4^ cells/well and cultured at 37 °C for 24 h. Then, the cells were washed with PBS and incubated with L, TL, PL and PTL diluted with FBS-free DMEM medium (the concentration of coumarin-6 was 100 ng/mL) for 1 h at 37 °C. After 1 h, the medium was removed and cells were washed three times with PBS. Then, the cells were fixed with 4% paraformaldehyde for 15 min. After fixing, the cells were washed three times with PBS and then dyed with DAPI (2 ug/ml) for 15 min followed by washing three times. Then, the cover slips were took out and covered on the slides. The fluorescent images of the cells were analyzed using a A1 + R confocal microscope (Nikon, Japan).

### Effects of free PCM and TAT peptides on uptake

MCs were seeded at a density of 30 × 10^4^ cells/well in 6-well plates and cultured overnight at 37 °C. Before incubating with TL and PL, the cells were pre-incubated with free TAT (0.5 mg/ml) and PCM (0.5 mg/ml) peptides were diluted with FBS-free DMEM medium, respectively, for 30 min at 37 °C. Then, the peptide suspensions were removed and the cells were washed with PBS for three times. The cells were then treated with TL and PL, and diluted with FBS-free DMEM medium (the concentration of coumarin-6 was 200 ng/mL) for 1 h at 37 °C. Next, the cells were washed and harvested for flow cytometry measurements.

## *In vivo* distribution of L, TL, PL and PTL

### Frozen sections of the mouse heart

A total of 13 Kunming mice (6 male and 7 female, 15 ± 2 g) were randomly divided into five groups (three mice in each experimental group, and one mouse in control group). 200 μl of L, TL, PL and PTL were injected into the tail vein of mice at a coumarin-6 dose of 0.1 mg/kg. The control group was injected in 200 μl PBS buffer. One hour later, all the mice were narcotized with 3% pentobarbital sodium (30 mg/kg). Then, the hearts were perfused with normal saline and removed rapidly. The hearts were then sliced into contiguous sections on coronal plane (10 μm in thickness) by a CM1860 freezing microtome (Leica, Germany). The sections were fixed with cold acetone for 15 min and dyed with DAPI for 15 min to mark nucleus at room temperature. Then, the sections were washed three times with cold PBS and sealed with 4% glycerol. The distribution of various liposomes in mice hearts were observed by a A1 + R confocal microscope (Nikon, Japan).

### High-performance liquid chromatography assay

A total of 12 Kunming mice (six male and six female, 15 ± 2 g) were randomly divided into four groups (three mice in each group). 200 μl of L, TL, PL and PTL were injected into the tail vein of mice at a coumarin-6 dose of 0.1 mg/kg. One hour later, all the mice were narcotized with injecting 3% pentobarbital sodium (30 mg/kg). Then, the hearts were perfused with normal saline and removed rapidly. Then, the hearts were snipped and ground to obtain heart homogenate. 200 μl of homogenate was transferred to a centrifuge tube and 10 μl coumarin-7 (1 ug/ml) was added to the homogenate. Then, 1 ml hexane was added to the homogenate to extract coumarin-6 and coumarin-7, followed by vortexing, ultrasonic and centrifuging at 8000 *g* for 5 min. Then, the supernatant was transferred to another centrifuge tube and solvent was evaporated at room temperature. 200 μl of methanol was added to the centrifuge tube to dissolve the residue and centrifuged at 10 000 *g* for 10 min. Finally, the supernatant was obtained and was analyzed by an Agilent 1260 HPLC System that contains a fluorescence detector (Santa Clara, CA). The mobile phase consisted of methanol and ultrapure water (v/v = 95:5). Separation was carried out at 37 °C using a reverse-phase C_18_ column (Diamonsil TM, 200 × 4.6 mm, 5 μm, Dikma Tech Co. Ltd., Beijing, China). The excitation and emission wavelength was 465 nm and 502 nm, and a flow rate of 1.0 mL/min was employed.

### Statistical analysis

Results were shown as mean ± SD. Comparisons were performed using unpaired Student’s *t*-test and one-way ANOVA and then by the Student–Newman–Keuls test. A *p* value < 0.05 was considered statistically significant.

## Results

### Characterization of the liposomes

**Figure 1.**
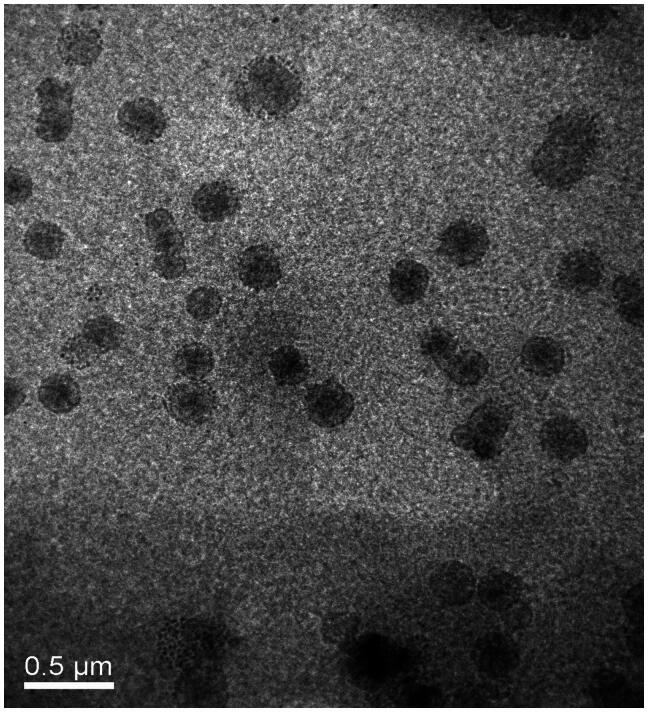
The transmission electron microscope (TEM) photograph of PTL (Scale bar = 0.5 μm).

Particle size, zeta potential and entrapment efficiency of L, TL, PL and PTL are shown in Table 1. The particle size of coumarin-6 loaded L was around 110 nm and were approximately 111–115 nm after PCM or/and TAT modified. The value of the zeta potential of liposomes had a negative charge of about −10 mV to −15 mV and the EE values were all higher than 80%. In addition, transmission electron microscope (TEM) photograph showed that TPL was spherical and regularly shaped (Figure 1).

**Figure 2.**
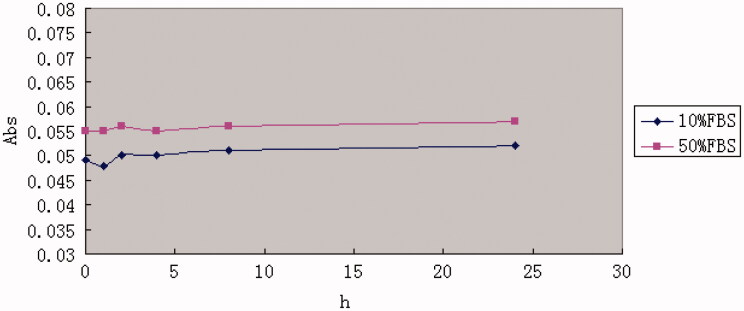
The variations in turbidity (represented by absorbance) of PTL in 10% FBS and 50% FBS. The results were represented as means ± SD (*n* = 3).

**Figure 3.**
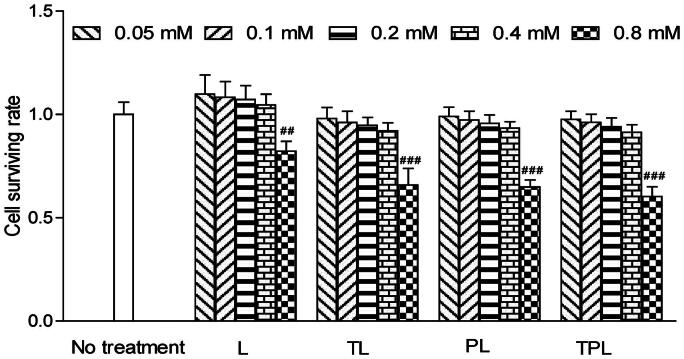
Cell viabilities in MCs with the treatments of different liposomes with different lipid concentration from 0.05 to 0.8 mM. The lipid concentrations were from 0.05 to 0.8 mM. The results are represented as means ± SD (*n* = 3). ##*p* < 0.01; ###*p* < 0.001 versus no treatment.

**Table 1.** Results of the particle size, PDI, zeta-potential and encapsulation efficiency of various liposomes.

Samples	Size/nm	PDI	Zeta/mV	EE/%
L	108.7 ± 0.78	0.20 ± 0.03	−15.3 ± 0.78	89.3 ± 2.6
PL	111.6 ± 0.42	0.21 ± 0.01	−10.3 ± 1.48	85.8 ± 2.2
TL	112.5 ± 1.06	0.18 ± 0.01	−12.4 ± 0.35	86.2 ± 2.7
PTL	114.4 ± 2.69	0.19 ± 0.02	−13.5 ± 1.84	84.6 ± 3.2

Data were represented as the mean ± SD (*n* = 3).

### *In vitro* stability of PTL

As shown in Table 2, the particle size, zeta-potential, polydispersity index (PDI) and encapsulation efficiency of PTL did not show any drastic increase, suggesting that PTL was stable at 4 °C in PBS, pH 7.4 for 30 d. Besides, the varying curve of absorbance shown in Figure 2 demonstrated that PTL had no significant changes in 10% FBS and 50% FBS.

**Table 2.** The variations in particle size, PDI, zeta-potential and encapsulation efficiency of PTL in 4 °C PBS between day 0 and day 30.

Day	Size/nm	Zeta/mV	PDI	EE/%
0	114.4 ± 2.69	−13.5 ± 1.84	0.19 ± 0.02	84.6 ± 3.2
30	117.1 ± 2.94	−15.6 ± 2.93	0.24 ± 0.05	83.7 ± 3.5

Data were represented as the mean ± SD (*n* = 3).

### Liposomal cytotoxicity

The cytotoxicity of the various liposomes was evaluated with a series of liposome concentration of 0.05, 0.1, 0.2, 0.4 and 0.8 mM (Figure 3). Cell viability in the serum-free culture as well as a control was set at 100%. As for the relative viability of MCs, there were no obvious changes in the groups treated with the concentration less than 0.4 mM. However, there was a significant reduction of cell viability in the group treated with a concentration greater than 0.8 mM. Accordingly, the concentration of liposomes was controlled less than 0.8 mM in *in vitro* experiments.

### Qualitative and quantitative of cellular uptake *in vitro*

**Figure 4.**
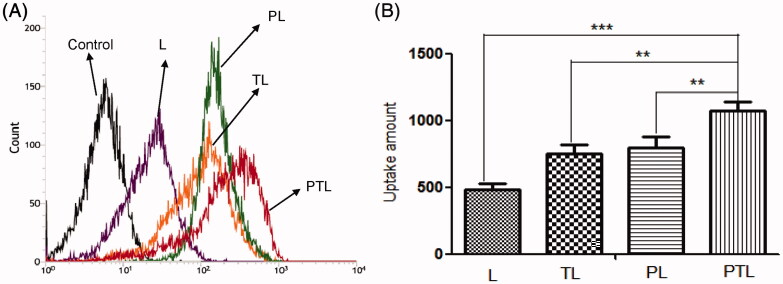
Cellular uptake of L, TL, PL and PTL on MCs. (A) The uptake of four kinds of liposomes loaded coumarin-6 by the MCs were determined by flow cytometry. (B) The cellular uptake was investigated quantitatively. The results were represented as means ± SD (*n* = 3). **p < 0.01; ***p < 0.001 versus PTL.

Data from MCs uptake assay showed that the mean fluorescence intensity of coumarin-6 were 487.7 for L, 753.3 for TL, 799 for PL and 1079 for PTL (Figure 4). Compared with L, the data showed that uptakes of TL, PL and PTL by MCs *in vitro* were increased to 154.4%, 163.8% and 200.7%, respectively. The results demonstrated that the liposomes after modification with TAT or PCM were able to be internalized by MCs more easily, and dual-ligands liposome PTL possessed the highest cellular uptake rate in MCs, which might be the effect of the synergistic effect of two ligands.

**Figure 5.**
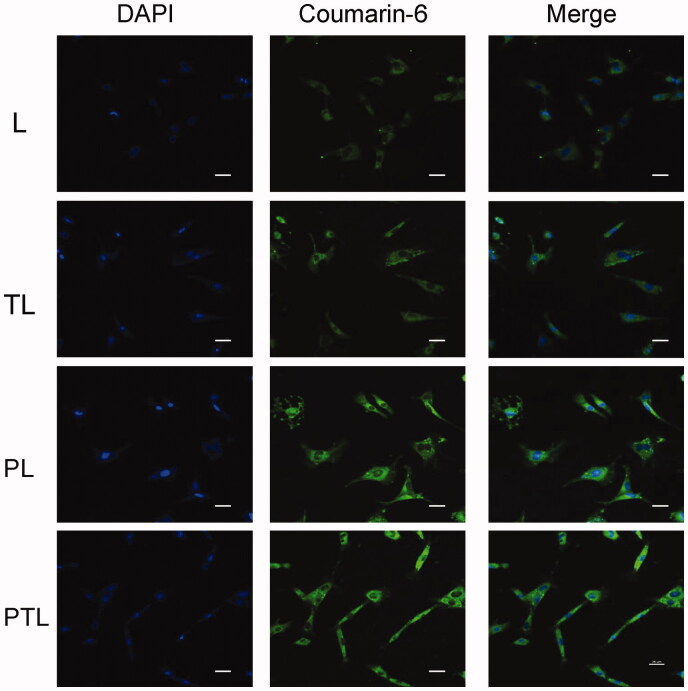
Cellular uptake of L, TL, PL and PTL on MCs. Four kinds of liposomes loaded coumarin-6 showed green autofluorescence and their uptake by the MCs recorded by confocal microscopy. Cell nuclei were stained blue with DAPI. Bar = 20 μm.

The distribution of various liposomes containing coumarin-6 in MCs is shown in Figure 5. Coumarin-6 displayed green fluorescence, DAPI combined with DNA to label the nucleus and showed blue fluorescence. After incubation with L, TL, PL and PTL, the coumarin-6 fluorescence was coincident with DAPI fluorescence, which showed that the fluorescence of coumarin-6 was mainly distributed in the cytoplasm. The result showed that the green fluorescence intensity of PTL was strongest, followed by PL and TL, the green fluorescence intensity of L is weakest.

### Competition assay of PCM and TAT peptides

**Figure 6.**
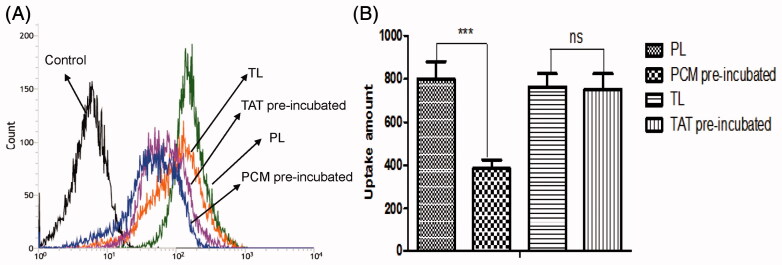
Cellular uptake of TL and PL on MCs pre-incubated by free TAT or PCM peptides. (A) The uptake of two kinds of liposomes loaded coumarin-6 by the MCs were determined by flow cytometry. (B) The cellular uptake was investigated quantitatively. The results were represented as means ± SD (*n* = 3). ***p < 0.001, ns: no significant difference.

The effects of free PCM and TAT peptides on the uptake of TL and PL by MCs were measured by flow cytometry. As shown in Figure 6, the mean fluorescence intensity of coumarin-6 uptake of PL pre-incubated by PCM was 385, whereas the uptake of PL without incubated by PCM was 799. The ratio of the two was 2.07. Besides, the mean fluorescence intensity of coumarin-6 uptake of TL pre-incubated by TAT was 753.3 and the uptake of TL without incubated by TAT was 763. The ratio of the two was 1.01. The result confirmed that PCM receptors could be saturated by PCM and endocytosis mediated by TAT would not be affected by free TAT peptides.

### Qualitative and quantitative of cellular uptake *in vivo*

**Figure 7.**
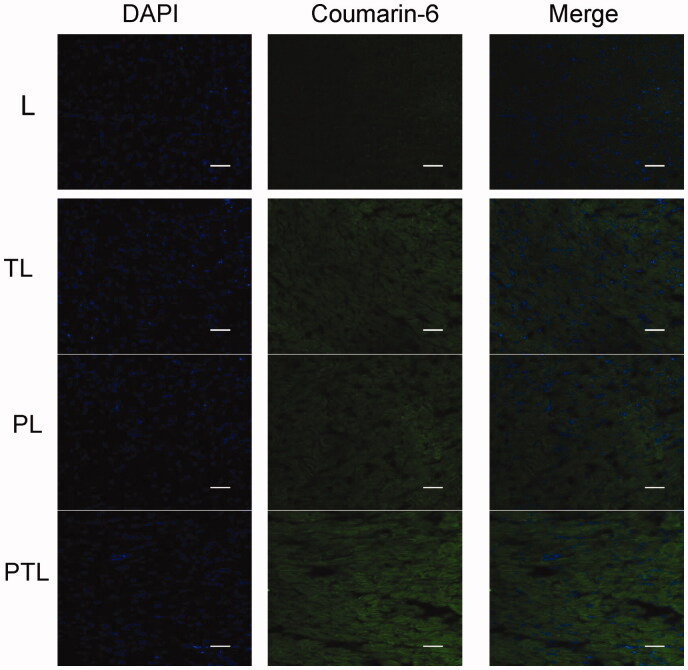
The confocal microscopic images of frozen sections of mouse heart. Four kinds of liposomes loaded coumarin-6 showed green autofluorescence and their uptake by the myocardium recorded by confocal microscopy. Cell nuclei were stained blue with DAPI. Bar = 100 μm.

Figure 7 shows the confocal microscopic images of frozen sections of mouse heart. Coumarin-6 was mainly distributed in the cytoplasm and displayed green fluorescence. DAPI-labeled nuclei showed blue fluorescence. The images showed that PTL exhibited a more intense fluorescence than L, TL and PL. The results were consistent with the results of cellular uptake study, indicating that PTL was efficiently internalized by cardiomyocyte under the existence of both PCM and TAT.

**Figure 8.**
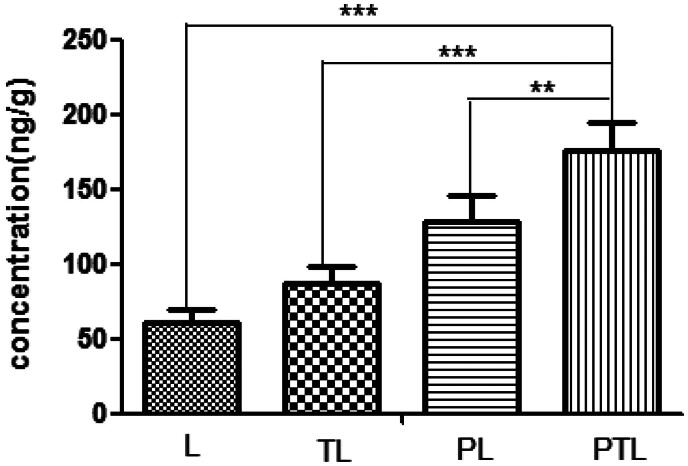
The amount of L, TL, PL and PTL accumulated into the mouse heart detected by high performance liquid chromatography assay (HPLC). The results were expressed as the weight ratio of coumarin-6 and heart (ng/g) and represented as means ± SD (*n* = 3). **p < 0.01; ***p < 0.001 versus PTL.

The amount of various liposomes accumulated into the heart was detected by HPLC and expressed as the weight ratio of coumarin-6 and heart (ng/g). As shown in Figure 8, the total amount of PTL gathered to the heart is 2.9 times as much as L (*p* < 0.001), 2.0 times of TL (*p* < 0.01) and 1.4 times of PL (*p* < 0.01). These results suggested that PCM and TAT co-modified liposome had a better myocardium targeting ability.

## Discussion

In this study, a novel drug carrier of PCM and TAT co-modified liposome (PTL) was developed for the myocardium delivery. It was the first attempt to prepare dual-ligand liposome by using PCM and TAT to construct drug deliver vector for myocardium targeting. PCM and TAT co-modified liposome would increase the myocardium uptake *in vitro* and *in vivo*. In addition, the unmodified liposome (L), PCM-modified liposome (PL) and TAT-modified liposome (TL) were used as the control to reveal the superior targeting ability of co-modified liposome (PTL).

Several myocardium-affinitive ligands were used to combine with liposomes for myocardial targeting. Dasa et al. (2015) found that the 7-mer peptides were specific for cardiomyocytes using *in vivo* phage display methods and optical imaging approach. They further developed the 7-mer peptides-modified liposome to deliver a PARP-1 (poly [ADP-ribose] polymerase-1) inhibitor (AZ7379) to cardiomyocytes. These reports indicated that ligand-targeted liposome as a delivery system for the treatment of MI was extensively studied. PCM was chosen as the specific myocardium targeted ligand in this study. As shown in Figure 4 and Figure 5, PCM-modified liposomes have much better targeting ability than unmodified liposomes. Free PCM could inhibit the transport of liposomes modified with PCM ligands, which indicated that PCM-modified liposomes were transported into MCs through a PCM receptor-mediated endocytosis (Figure 6). However, there would be a decline in targeting efficiency with PCM single modification when receptor expression was downregulated and/or saturated (Warren et al., 1997; Sawant & Torchilin, 2012).

Cell-penetrating peptides (CPPs), short peptide sequences that had no saturation phenomenon, have been used for drug or gene delivery for some time (Joliot & Prochiantz, 2004; Banks et al., 2005). Among all the CPPs, the TAT which had good cell membrane penetration ability was already exploited for the delivery of nucleotides, proteins, drugs and genes into cells (Fittipaldi & Giacca, 2005; Koren, 2011). Therefore, we chose TAT to break the limitation of PCM receptor expression down-regulation and/or saturated. As shown in Figure 4 and Figure 5, TAT-modified liposomes were much more efficiently taken up by MCs than unmodified liposomes. In addition, data from competition assay (Figure 6) showed that free TAT has a weak effect on the uptake, which suggested there might be no receptor-mediated pathway in TAT-modified liposome.

The dual-modified delivery carrier has the advantages of improving the targeting accuracy and efficiency (Kluza et al., 2012), enhancing the drug uptake, improving the adhesion ability of the target point and the good blood flow stability (Ferrante et al., 2009). The increased uptake efficiency of the dual-ligand liposome was verified by flow cytometry (Figure 4), and the semi-quantitative result suggested that the dual-ligand liposome (PTL) demonstrated statistically significant difference compared with simple L and single-ligand L (TL and PL). After incubation with four different groups of liposomes, the intracellular distribution of liposomes was observed by the confocal microscopy (Figure 5). Green fluorescent (from the fluorescence of coumarin-6) signal and blue signal could be clearly observed from the cytoplasm and the nuclei of MCs. The fluorescence of coumarin-6 was mainly distributed in the cytoplasm indicated that liposome was mainly distributed in the cytoplasm. The result showed that the green fluorescence intensity of PTL was strongest, which demonstrated that dual-ligand liposome had the best uptake efficiency. The *in vivo* targeting effect of liposome was evidenced by confocal imaging of frozen section (Figure 7) and HPLC assay (Figure 8), the results were consistent with the results of cellular uptake study.

The connecting methods of the ligand and the liposome include organic phase reaction (Katanasaka et al., 2008), aqueous reaction (Steenpass et al., 2006) and insertion method (Ishida et al.,^1999^). In this study, we applied insertion method to prepare liposome. PCM and TAT were connected covalently to the distal end of DSPE-PEG2000-MAL micellar and then incubated with liposome in phosphate buffer. The particle sizes of coumarin-6 loaded liposomes were about 110 nm to 115 nm and the values of the zeta potential were −10 mV to −15 mV. There were no significant differences in the particle size and zeta potential among simple liposome and PCM or/and TAT-modified liposome. Thus, the connections of PCM and TAT did not significantly alter the particle size and zeta potential of various liposomes. *In vitro* stability experiment suggested liposomes were stable at a pH 7.4 PBS and FBS. In addition, the cytotoxicity experiment showed there were no obvious changes for the relative viability of MCs in the different liposomes at a concentration less than 0.8 mM.

In conclusion, the novel dual-ligand liposome delivery system developed in present paper had the possibility to provide better cardiomyocytes targeting ability. It would be able to deliver therapeutic drugs to the myocardium effectively for the treatment of myocardial ischemia.
